# Famotidine activates the vagus nerve inflammatory reflex to attenuate cytokine storm

**DOI:** 10.21203/rs.3.rs-1493296/v1

**Published:** 2022-04-11

**Authors:** huan yang, Sam J George, Dane Thompson, Harold A Silverman, Tea Tsaava, Aisling Tynan, Valentin A Pavlov, Eric Chang, Ulf Andersson, Michael Brines, Sangeeta S Chavan, Kevin J Tracey

**Affiliations:** Feinstein Institute for Medical Research; Feinstein Institute for Medical Research Fertility Research Laboratory: Northwell Health Feinstein Institutes for Medical Research; Feinstein Institute for Medical Research Fertility Research Laboratory: Northwell Health Feinstein Institutes for Medical Research; Feinstein Institute for Medical Research Fertility Research Laboratory: Northwell Health Feinstein Institutes for Medical Research; Feinstein Institute for Medical Research Fertility Research Laboratory: Northwell Health Feinstein Institutes for Medical Research; Feinstein Institute for Medical Research Fertility Research Laboratory: Northwell Health Feinstein Institutes for Medical Research; Feinstein Institute for Medical Research Fertility Research Laboratory: Northwell Health Feinstein Institutes for Medical Research; Feinstein Institute for Medical Research Fertility Research Laboratory: Northwell Health Feinstein Institutes for Medical Research; Karolinska Institutet; Feinstein Institute for Medical Research Fertility Research Laboratory: Northwell Health Feinstein Institutes for Medical Research; Feinstein Institute for Medical Research Fertility Research Laboratory: Northwell Health Feinstein Institutes for Medical Research; Feinstein Institute for Medical Research Fertility Research Laboratory: Northwell Health Feinstein Institutes for Medical Research

**Keywords:** histamine, histamine receptors, vagus nerve signaling, cholinergic anti-inflammatory pathway

## Abstract

**Background.:**

Severe COVID-19 is characterized by pro-inflammatory cytokine release syndrome (cytokine storm) which causes high morbidity and mortality. Recent observational and clinical studies suggest famotidine, a histamine 2 receptor (H2R) antagonist widely used to treat gastroesophageal reflux disease, attenuates the clinical course of COVID-19. Because evidence is lacking for a direct antiviral activity of famotidine, a proposed mechanism of action is blocking the effects of histamine released by mast cells. Here we hypothesized that famotidine activates the inflammatory reflex, a brain-integrated vagus nerve mechanism which inhibits inflammation via alpha 7 nicotinic acetylcholine receptor (α7nAChR) signal transduction, to prevent cytokine storm.

**Methods.:**

The potential anti-inflammatory effects of famotidine and other H2R antagonists was assessed in mice exposed to lipopolysaccharide (LPS)-induced cytokine storm. As the inflammatory reflex is integrated and can be stimulated in the brain, and H2R antagonists penetrate the blood brain barrier poorly, famotidine was administered by intracerebroventricular (ICV) or intraperitoneal (IP) routes.

**Results.:**

Famotidine administered IP significantly reduced serum and splenic LPS-stimulated tumor necrosis factor α and interleukin-6 concentrations, significantly improving survival. The effects of ICV famotidine were significantly more potent as compared to the peripheral route. Mice lacking mast cells by genetic deletion also responded to famotidine, indicating the anti-inflammatory effects are not mast cell dependent. Either bilateral sub-diaphragmatic vagotomy or genetic knock-out of α7nAChR abolished the anti-inflammatory effects of famotidine, indicating the inflammatory reflex as famotidine’s mechanism of action. While the structurally similar H2R antagonist tiotidine displayed equivalent anti-inflammatory activity, the H2R antagonists cimetidine or ranitidine were ineffective even at very high dosages.

**Conclusions.:**

These observations reveal a previously unidentified vagus nerve-dependent anti-inflammatory effect of famotidine in the setting of cytokine storm which is not replicated by high dosages of other H2R antagonists in clinical use. Because famotidine is more potent when administered intrathecally, these findings are also consistent with a primarily central nervous system mechanism of action.

## Introduction

The recent emergence and rapid spread of the SARS-CoV-2 virus has resulted in a catastrophic COVID-19 pandemic characterized by high morbidity and mortality. Emerging evidence suggests that dysregulated immune responses play a critical role in fueling a cascade of excessive inflammation which is a key feature of severe COVID-19 (1–4). The hyperactive inflammatory response elicited by severe acute respiratory syndrome coronavirus 2 (SARS-CoV-2) frequently culminates in the cytokine release syndrome (cytokine storm). Critical features of cytokine storm involve the infiltration, expansion and activation of monocytes and macrophages and other immune competent cells with the consequent excessive production of pro-inflammatory cytokines and chemokines, e.g., TNF and IL-6 (1, 4), which drive the clinical course, e.g., causing acute respiratory distress syndrome (3).

Many drugs have been evaluated as potential treatments of the inflammatory components of COVID-19, but few compounds have improved outcome. Among these, anti-IL-6 biologicals and high dose glucocorticoids may benefit patients undergoing cytokine storm (4, 5). However, anti-IL-6 therapy targets only a single component of a complicated pro-inflammatory cascade and incompletely blocks inflammatory processes. Although glucocorticoids more broadly suppress the inflammatory response, their use is associated with impaired clearance of viral load, as well as associated serious potential adverse effects in many tissues (6). Therefore, a search for additional pharmacological approaches to inhibit lethal cytokine storm is warranted.

Several observational studies of COVID-19 patients indicate famotidine, a selective histamine H2 receptor antagonist widely used to treat gastroesophageal reflux disease, improves survival in hospitalized patients and ameliorates symptoms in non-hospitalized patients (7, 8) when administered at a high dose. A recent randomized placebo controlled double blind clinical trial has shown that high dose oral famotidine administered to symptomatic, non-hospitalized COVID-19 patients accelerates the suppression of interferon γ and improves symptom resolution (9). Although these and other clinical findings are potentially important, enthusiasm for using famotidine has been hampered because the molecular mechanism of action underlying famotidine’s beneficial effects remains enigmatic.

The chemical structure of famotidine led some to theorize it might interfere with the viral proteases required for SARS-CoV-2 replication, but *in silico* studies indicate famotidine does not inhibit either SARS-CoV-2 proteases or SARS-CoV-2 viral replication (10–12). Pharmacologically, famotidine is classified as an inverse agonist, i.e., a competitive antagonist of histamine-induced receptor activation, while also decreasing baseline H2 receptor signaling (13). Famotidine has been implicated as an inhibitor of histamine-induced toll-like receptor 3-mediated inflammatory signaling in SARS-CoV-2 infected cells in vitro (14). Famotidine may also act through other on-target histaminergic mechanisms via gastric acid reduction (12, 15) or immune cell generation of pro-inflammatory mediators (12, 16, 17). Additionally, potentially relevant off-target effects of famotidine include scavenging reactive oxygen radicals, especially the hydroxyl ion (18–22), which may reduce secondary inflammation and damage. Accordingly, the mechanistic basis for the beneficial effects of famotidine in COVID remain unidentified. Because H2 antagonists are polar, and do not readily pass the blood brain barrier, we reasoned here that the high dose requirements for famotidine efficacy might be attributable to targeting in the central nervous system (CNS), which abundantly expresses all four histamine receptors (13, 23).

The inflammatory reflex is a vagus nerve mediated homeostatic mechanism which inhibits cytokine storm. Vagus nerve signals arising in the brain stem attenuate inflammation by cholinergic signal transduction which activates the α7 nicotinic acetylcholine receptor (α7nAChR) expressed on cytokine producing cells and thereby inhibiting cytokine release. Since H2R is expressed in the central nervous system, we used a murine model of cytokine storm to assess the role of famotidine in stimulating the inflammatory reflex (24) (25). The results indicate that famotidine inhibits endotoxin-induced cytokine storm and improves survival via a vagus nerve dependent, but histamine H2 receptor-independent mechanism.

## Materials and Methods

Lipopolysaccharide (*E. coli*. 0111:B4), histamine (Cat # H7125), ranitidine (Cat # R101), famotidine (Cat # F6889) and cimetidine (Cat # C4522) were purchased from Sigma (St. Louis, MO). Famotidine (iv vial, 20 mg/2ml) was from APP Pharmaceutical LLC (Schaumburg, IL). Tiotidine (Cat # 0826) was from Tocris Bioscience (Minneapolis, MN). Fetal bovine serum was obtained from Gibco BRL (Carlsbad, CA). Glutamine was from Biowhittaker Inc., (Walkersville, MD). Thioglycollate medium was purchased from Becton Dickinson Co., (Sparks, MD). Protease inhibitor minitablets (Cat # A32953) were form Thermo Fisher Scientific Inc., (Waltham, MA).

## Cell culture

Murine macrophage-like RAW 264.7 cells were obtained from American Type Culture Collection (Rockville, MD). RAW 264.7 cells were cultured in DMEM or in RPMI 1640 medium, respectively, supplemented with 10% fetal bovine serum, 100 U/ml penicillin and 100 μg/ml streptomycin. Thioglycolate-elicited peritoneal macrophages were maintained in RPMI 1640 medium before use. Cells in culture plates were used at 90% confluence. Stimulation to cells was carried out in serum-free Opti-MEM I medium (Life Technologies, Carlsbad, CA).

Primary mouse thioglycollate-elicited macrophages were obtained as previously described (26). Briefly, each mouse was injected intraperitoneally with 2 ml of thioglycollate broth (4%). Two days later, mice were euthanized and 5 ml of 11.6% sterile sucrose was injected into the peritoneal cavity. Lavage fluid from peritoneal cavity (containing macrophages) was collected by using BD insyte autoguard (BD bioscience, San Jose, CA). Cells were then passed through a strainer (BD Falcon, Franklin Lakes, NJ) to remove debris. After washing with RPMI medium, cells were suspended in RPMI 1640 medium supplemented with 10% heat-inactivated fetal bovine serum (FBS), 2 mM glutamine and 100 U/mL penicillin, 100 μg/mL streptomycin. Cells were seeded in 96-well Primaria tissue culture dishes (Life Technologies, Grand Island, NY) and allowed to rest for 18–24 h at 37°C incubator. All treatments were carried out in serum free Opti-MEM I medium (Life Technologies).

## Animal experiments

### Animals

Male C57BL/6, α7 nicotinic acetylcholine receptor knockout (stock # B6.129S7-Chrna7<tm1Bay>/J) mice (8–12 weeks old) or mast cell deficient *Kit*^*W-sh*^*/Kit*^*W-sh*^ sash mice (stock # 030764, C57BL/6J-congenic *Kit*^*W-sh*^) (all 8–12 weeks old), were obtained from the Jackson Laboratories (Bar Harbor, ME) and acclimated for at least 1 week before conducting experiments. All animal procedures were approved by the Feinstein Institutes for Medical Research Institutional Animal Care and Use Committee (IACUC, protocol #2016–028). Mice were housed in the Center for Comparative Physiology of the Feinstein Institutes for Medical Research under standard temperature, light and dark cycle conditions.

### Vagotomy and transgastric pyloric dilation surgery

Surgery for bilateral sub-diaphragmatic vagotomy was performed following the published methods (27). Mice were anesthetized with isoflurane (2%) and oxygen (1.25 L/min) and placed in a supine position and a midline celiotomy was made. Visceral organs were moved to the right, exposing the esophagus. Left and right vagus nerves were identified and transected just inferior to the diaphragm. The abdomen was then closed with sutures and/or staples.

Transgastric pyloric dilation surgery. This procedure was performed following bilateral sub-diaphragmatic vagotomy surgery in order to reduce the side effects of stomach distension following vagotomy, as vagus nerve controls stomach emptying. Following induction of anesthesia with intramuscular injection of a mixture of ketamine (50 mg/kg) and xylazine (10 mg/kg), the mouse was fixed in supine position. A midline celiotomy was made and carried superiorly to the xyphoid process in order to ensure the best visualization of the foregut structures. A small retractor (2.75” Alm Retractor, Cat# RS6510, Roboz Surgical Instrument Co., Gaithersburg, MD) was placed in the celiotomy to provide exposure. The anterior surface of the stomach was brought into the field of view with gentle, caudal retraction of the transverse colon. A space on the greater curvature of the stomach, without obvious surface vessels or mesocolic attachment, approximately 2 cm from the pylorus was identified and stabilized with a forceps. A 0.5 cm gastrotomy was made and the pylorus was accessed through the stomach for serial dilation, beginning with a 2 mm probe and concluding with a 4 mm probe (coated in water-soluble lubricant, Surgilube, HR Pharmaceuticals, York, PA), progressing in 0.5 mm increments (Cat #S1599–7002, Garrett Vascular Dilator precision medical device Inc., Hawthorne, NY). Once the probe is through the muscle, the probe was left in place for one minute to ensure adequate dilation. Following dilation, a 5–0 Vicryl suture was used to close the gastrotomy. Following surgery, warm saline (1ml) was administered subcutaneously, and mice were placed in a warm cage to recover. Liquid diet was provided for 3–5 days post-surgery.

### Intracerebroventricular (ICV) Injection

The procedure was performed following published method (28). Mice were anesthetized with a mixture of ketamine (50 mg/kg) and xylazine (10 mg/kg) and placed in a stereotactic head frame using ear bars (Stoelting Co. Wood Dale, IL). The incisor bar was adjusted until the plane defined by the lambda, and bregma was parallel to the base plate. A midline skin incision on the brain was made and the fascia is separated. The needle of a Hamilton syringe (25 μl) was stereo-tactically guided into the right lateral ventricle (1.2 mm lateral to the right, 0.6 mm posterior, 2.1 mm depth/ventral) at a rate of 1mm/min. Once the needle of the Hamilton syringe was inserted, test compound or vehicle (in 5 μl volume) was injected into the right lateral ventricle (1μl/min) and withdrawal of the syringe was performed slowly (4–5 minutes) to prevent backflow. The incision was sutured shut with 5–0 vicryl suture and 1mL of saline was injected subcutaneously. Mice were allowed to recover in a clean cage near a heat lamp.

### Electrophysiological recording

The vagus nerve recordings were performed as described previously (29, 30). Briefly, C57BL/6 mice were anesthetized using isoflurane at 2.5% in 100% oxygen at a flow rate of 1 L/min, and maintained at 1.5 % isoflurane on a heating pad to keep the core body temperature around 37°C. In prone position the animal’s head was fixed in a stereotactic frame. The sterile 26G stainless steel cannula (Plastics One, Inc.) was incerted into the left lateral ventricle using the coordinates from Paxinos and Franklin atlas and secured using Vetbond (Butler Schein, Dublin, OH). The animals were then repositioned in the left lateral recumbent, and the vagus nerve was isolated from the caroted sheath. Platinum-irridium cuff electrodes (CorTec, Freiburg, Germany) were placed on the nerve with reference electrode set in the neck muscle. Electrophysiological signals were recorded using a Plexon data-acquisition system (OmniPlex, Plexon, Inc.) at 40kHz sampling rate. Vagus nerve activity was aquired for 30 minutes prior to and post- ICV administration of either 5uL 0.4mg/mL Famotidine or normal Saline (BD, Franklin Lakes, NJ). The electrophysiological signals were analyzed using Spike2 software (CED, Cambridge, England) as described previously (30).

### LPS toxicity in mice

C57BL/6 or α7nAChR knockout mice (male, 8–12 weeks of age) had intraperitoneal injection of LPS (6–7 mg/kg) and treated with test compound or vehicle control administered IP or ICV either immediately after LPS injection or, in some experiments, prior to LPS injection at times indicated in the text. Two and half or 6 hours later, mice were euthanized, serum and spleen were collected for analyses.

### Cytokine measurements

Mouse TNF, IL-6, IL-1β, CXCL1 in cell culture supernatants or in samples of mouse serum or spleen were measured by commercially obtained ELISAs as per manufacturer’s instructions (R & D System Inc., Minneapolis, MN). Mouse splenic tissue was added to 1XPBS (400 μl per spleen) containing protease inhibitor and homogenized for 30 sec using Polytron homogenizer PT 3100 (VWR Scientific, Radnor, PA). The homogenate was then centrifuged at 2,000 rpm at 4C for 20 min. Supernatants were collected and transferred to a fresh tube. Protein concentration was measured using Bradford assay (Thermo Fisher).

### Statistical analysis

All data were analyzed using Prism 8.0 (GraphPad Prism) and were presented as means ± SEM. Differences between treatment groups were determined by student’s t test for comparison of two groups. For comparison of more than 2 groups, analysis was performed using one-way ANOVA followed by Tukey’s multiple comparisons test. In animal survival studies, differences between treatment groups were determined using 2-tailed Fisher’s exact test. P values less than 0.05 were considered statistically significant.

## Results

### Famotidine attenuates LPS-induced pro-inflammatory cytokine release and improves survival.

**Figure 1:**
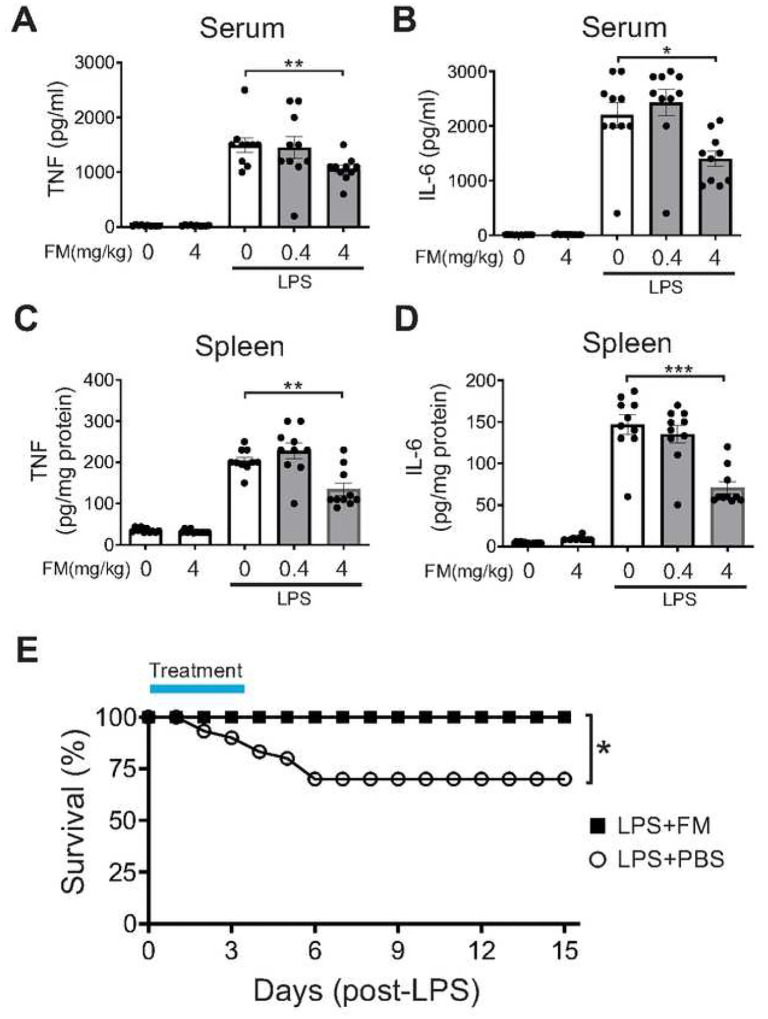
Famotidine attenuates lipopolysaccharide (LPS)-induced in ammatory responses in mice. A-D. Male C57BL/6 mice, 8–12 weeks of age, were injected with LPS (7 mg/kg) with or without famotidine (FM, 0.4 or 4 mg/kg, in 100 μl volume), intraperitoneally (IP) 30 min before LPS injection. Mice were euthanized 2.5 hours after LPS administration and serum and spleen TNF and IL-6 were measured. N=10 mice per group. *P<0.01, **P=0.001, ***P<0.001. E. Male C57BL/6 mice, 8–12 weeks old, were injected with LPS (6mg/kg, IP). Famotidine (FM, 4 mg/kg) or PBS (in 100 μl volume) were injected intraperitoneally twice a day for 3 days, survival was monitored for 2 weeks. N= 30 mice per group. *P=0.001 vs. LPS+PBS group.

To assess the potential role of famotidine in inflammation, we evaluated its effects on LPS-induced cytokine storm, a process driven in large part by the key pro-inflammatory cytokines TNF and IL-6 (24). C57BL/6 mice were administered famotidine (FM; 0.4 or 4 mg/kg, which corresponds (31) to a subtherapeutic versus therapeutic human equivalent dose of ~2 mg and ~20 mg respectively) or vehicle intraperitoneally 30 min prior to LPS (7 mg/kg). The animals were then euthanized at 2.5 hours post-LPS exposure, a timepoint appropriate for capture of both TNF and IL-6 release (32). Intraperitoneal administration of famotidine reduced LPS-induced TNF levels in a dose-dependent manner. The higher dose of famotidine significantly reduced LPS-induced elevated levels of serum and splenic TNF by ~ 40% and ~65% respectively and IL-6 by ~40% and ~50% (Fig. 1A–D). However, the suppressive effects of famotidine did not extend to IL-1β and CXCL1 within the serum and spleen at this timepoint, or 6 hours later (Suppl Fig. 1), suggesting a narrow anti-inflammatory target for famotidine’s early effects. Further, by 6 hours following LPS administration, famotidine no longer significantly altered serum or spleen IL-6 levels as compared to vehicle (Suppl Fig. 1D,G), indicating a short biological half-life for a single dose. As LPS elicits significant lethality over a period of several days, we also determined the effect of repeated administration of famotidine on the survival. In C57BL/6 mice subjected to LPS (6 mg/kg, intraperitoneally), treatment with famotidine (4 mg/kg, injected IP, twice daily for 3 days) significantly improved 2-week survival by ~30% compared to mice administered vehicle (Fig. 1E).

### Intracerebroventricular administration increases the potency of famotidine.

**Figure 2:**
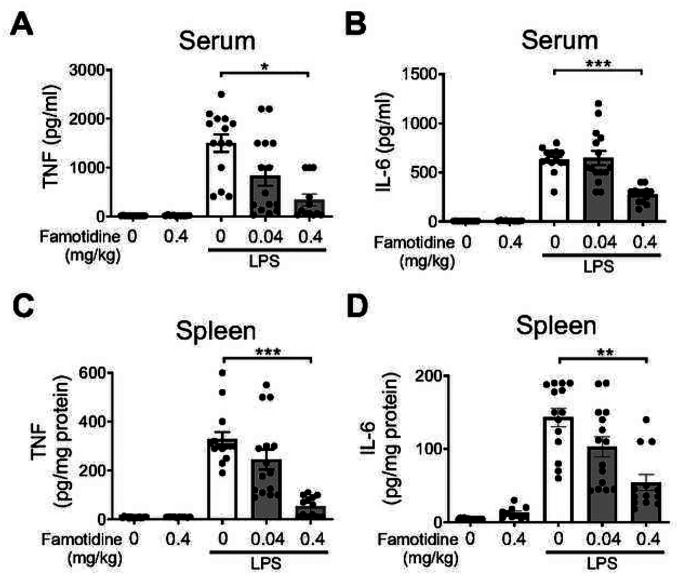
Famotidine is more potent when administered into the central nervous system. A-D. Famotidine (intracerebroventrical, ICV) injection attenuated LPS-induced systemic TNF and IL-6 release in mice. Male C57BL/6 mice had ICV injection of PBS or famotidine (0.04 or 0.4 mg/kg in 5 μl volume) 30 min before LPS. LPS was administered IP at 7 mg/kg. Mice were euthanized 2.5 hours post-LPS injection and serum and spleen were harvested for analyses. N=5 for normal group. N=10 for FM alone. N=13 or 14 for other groups. *P=0.0004. **P=0.0001. ***P<0.0001.

Human studies have shown that only ~9 % of an intravenous dose of famotidine pass through the intact blood brain barrier (33). Since the inflammatory response is coordinated in part within the CNS/brain (34), we determined whether central administration of famotidine increased efficacy. Accordingly, famotidine was administered ICV (0.04 or 0.4 mg/kg or vehicle, in 5 μl volume) 30 min prior to LPS (IP, 7 mg/kg) and pro-inflammatory cytokines assessed after 2.5 hours. Famotidine delivered via the ICV route required one tenth of the systemic route to block endotoxin-induced TNF levels in serum by 75% and in the spleen by 84% versus vehicle treated controls (Fig. 2A, C). Likewise, LPS-induced serum and spleen IL-6 were reduced in a famotidine dose-dependent manner (Fig. 2B, D). In confirmation of this effect, tiotidine, a H2R specific antagonist of chemical structure very similar to famotidine (13), resulted in identical cytokine suppression when administered ICV (Suppl Fig 2A–D). In contrast, administration of the first and second generation H2 antagonists cimetidine and ranitidine did not suppress LPS-associated pro-inflammatory cytokine production and did not improve survival (Suppl Fig 2E–P). Unlike peripheral administration, 6 hours after LPS exposure famotidine ICV injection significantly suppressed elevated serum levels of IL-6, but not IL-1β (Suppl Fig. 3A–B).

### Major cellular targets of LPS are not directly inhibited by famotidine.

**Figure 3:**
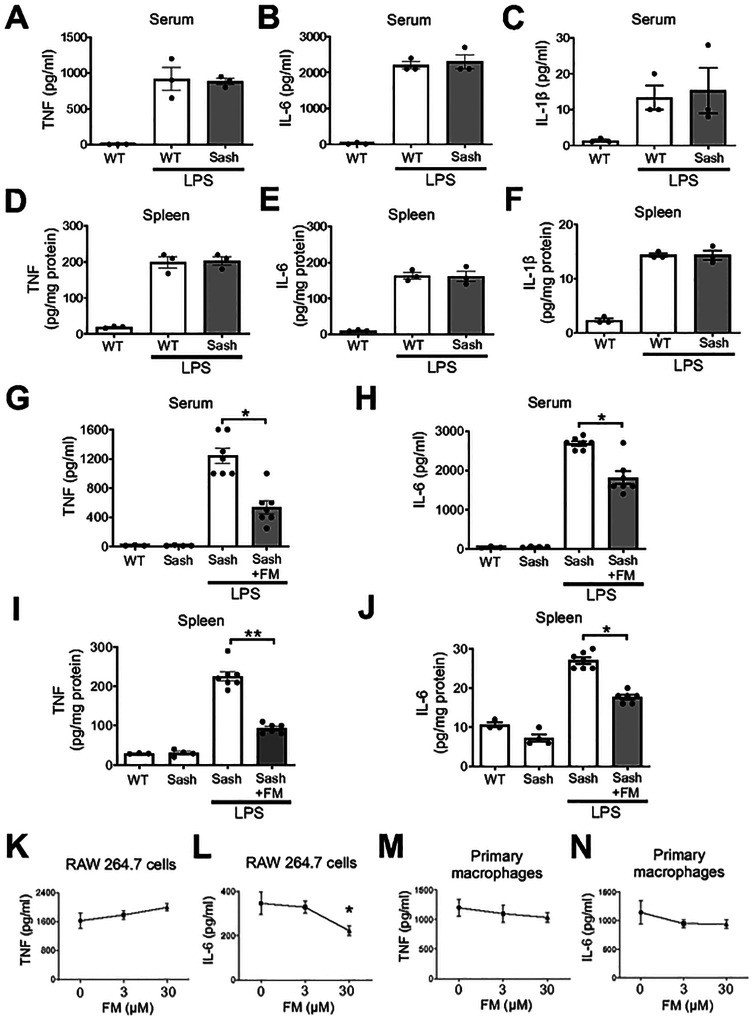
Mast cell de cient KitW-sh/KitW-sh sash mice had similar LPS-induced TNF, IL-6, IL-1β response as wild type mice. Male wild type (WT) or “sash” mice received LPS injection (IP, 7mg/kg) and were euthanized 2.5 hours post-LPS injection. Serum and spleen TNF, IL-6 and IL-1β were measured. N=3. G-J Famotidine, administered ICV, reduced LPS-induced TNF and IL-6 release in mast cell de cient KitW-sh/KitW-sh sash mice at 2.5 hours post LPS exposure. Male KitW-sh/KitW-sh sash mice received famotidine or PBS (0.4 mg/kg, in 5 μl volume) administered ICV at 30 min prior to LPS injection (IP, 7mg/kg). Mice were euthanized 2.5 hours post-LPS injection and serum TNF and IL-6 were measured. N=3 for normal wild type, n= 4 for normal “sash”, 5 for LPS, 5 or 7 for LPS+ famotidine group. *P<0.001. **P<0.0001. K-N. RAW 264.7 cells (K-L) or thioglycollate-elicited mouse primary peritoneal macrophages (M-N) in 96-well culture plates were stimulated with LPS (0.4ng/ml) in combination with various amounts of famotidine for 16 hours. TNF (K,M) and IL-6 (L,N) released in the supernatants were measured. n = 3–6 per treatment. *P<0.05 vs. LPS alone.

Mast cells have been hypothesized to drive severe COVID-19 (12, 16) and LPS activates mast cells to release key pro-inflammatory mediators, including IL-6 (35). To clarify whether mast cells contribute to famotidine’s anti-inflammatory effect, mast cell deficient *Kit*^*W-sh*^*/Kit*^*W-sh*^ sash mice were employed. This mouse carries a spontaneous *Kit* “sash” mutation, resulting in a universal lack of mast cells (36) and has been used successfully to study mast cell function (37, 38). *Kit*^*W-sh*^*/Kit*^*W-sh*^ sash mice exhibited similar cytokine (serum and splenic TNF, IL-6 and IL-1β) responses to LPS administration, as compared to wild type (Fig 3A–F). Administration of famotidine significantly suppressed LPS-induced both TNF and IL-6 release at 2.5 hours in the serum and within the spleen in these mice (Fig. 3G–J). Another key target of LPS activation is the macrophage. However, famotidine (0 to 30 μM) did not significantly inhibit LPS-induced TNF and IL-6 release in murine macrophage-like RAW 264.7 cells *in vitro* or in pro-inflammatory (thioglycollate-treated) primary mouse macrophages (Fig. 3K–N). Thus, famotidine does not directly antagonize LPS-induced cytokine release by either mast cells or macrophages.

### Famotidine activates the inflammatory reflex

**Figure 4:**
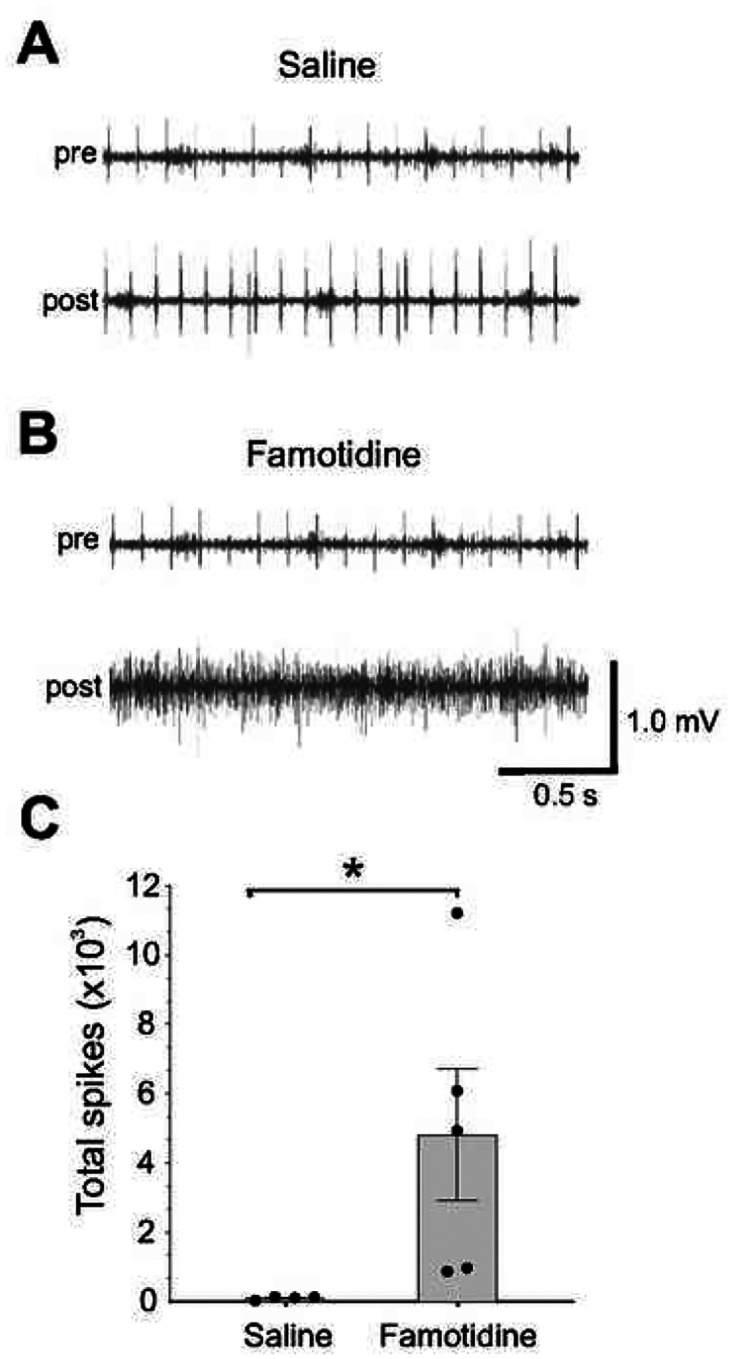
Famotidine activates the vagus nerve. A-B: Representative recordings of the vagus nerve signals in wild type mice pre- (baseline) and post- (A) saline or (B) famotidine administration ICV. Data is representative of 4 or 5 animals per group. Total spike count during recordings over the entire 4-minute post-ICV administration of saline and famotidine. (n=4 for saline and 5 for famotidine). C: *P = 0.029.

To determine whether ICV administration of famotidine induces efferent vagus nerve signaling, we recorded vagus nerve activity in real time following ICV famotidine administration. A micro cuff recording electrode was implanted on the cervical vagus nerve in wildtype mice prior to famotidine administration. ICV administration of famotidine significantly increases vagus nerve electrical activity, in contrast to no change in activity was observed following injection of saline (Fig. 4A–B). When comparing post injection vagus nerve activity, famotidine administration induces significantly more spikes than saline administration (Fig. 4C). Together, these data suggest that central administration of famotidine has a direct effect on vagus nerve signaling to the periphery.

**Figure 5:**
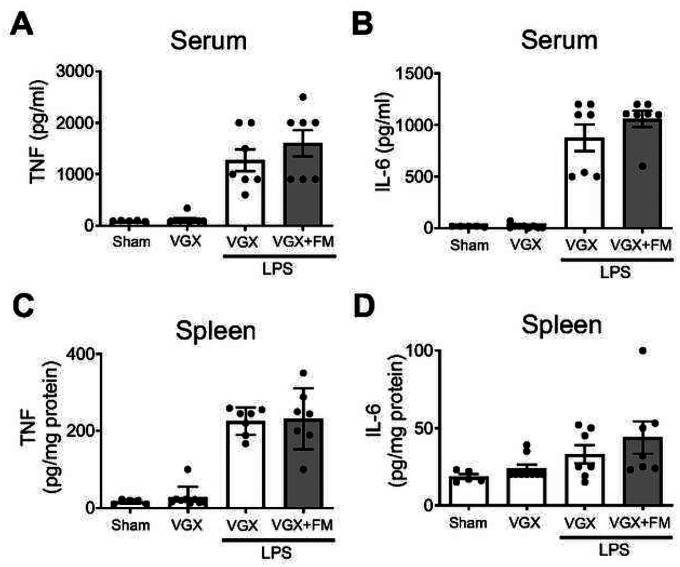
The suppressive effects of famotidine on LPS-induced pro-in ammatory cytokine release were abrogated in bilateral sub-diaphragm vagotomized mice. A-D. Male C57BL/6 mice had vagotomy (VGX; bilateral sub-diaphragmatic, with transgastric pyloric dilation to reduce stomach distension) and recovered for 7 days. Mice received famotidine (4 mg/kg) IP 30 min prior to LPS (IP, 7mg/kg). Mice were euthanized 2.5 hours later, serum and spleen TNF and IL-6 were measured. N=5 for normal; 7 per group for others.

**Figure 6:**
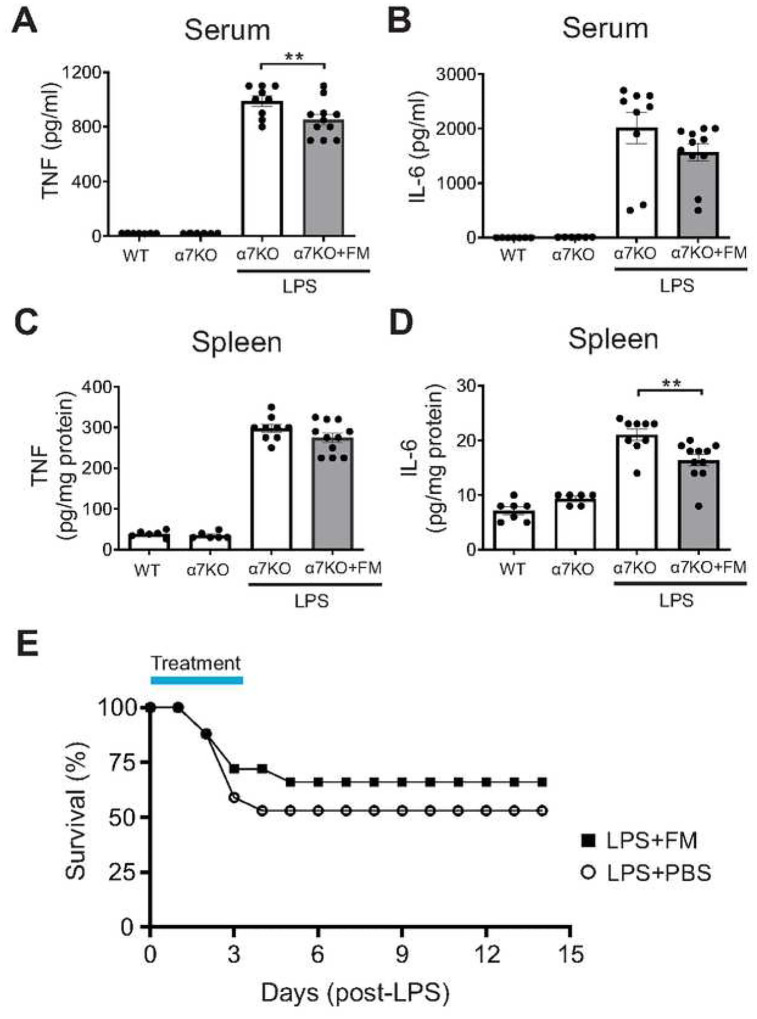
Suppressive effects of famotidine on LPS-induced TNF and IL-6 release were greatly attenuated and mortality increased in α7 nicotinic acetylcholine receptor knockout mice. A-D. Male C57BL/6 or α7nAChR knockout mice (α7KO; 8–12 weeks of age) received famotidine or PBS (0.4 mg/kg, in 5 μl volume) administered ICV at 30 min prior to LPS injection (IP, 7mg/kg). Mice were euthanized 2.5 hours post-LPS injection, serum and spleen TNF (B-C) and IL-6 (D-E) were measured. N=7 for wild type and 6 for α7nAChR KO controls, 9–11 per group for others. E. Male α7nAChR knockout mice, 8–12 weeks old, were injected with LPS (6mg/kg, IP). Famotidine (FM) or PBS (4 mg/kg, in 100 μl volume) were injected intraperitoneally twice a day for 3 days, survival was monitored for 2 weeks. N=17 for PBS control and 18 for FM mice per group.

The vagus nerve consists of both afferent (sensory) and efferent (motor) fibers which mediate anti-inflammatory activity via the inflammatory reflex which is integrated in the brain stem (39). To determine whether famotidine-mediated anti-inflammatory effects depend upon the vagus nerve, the effects of famotidine on LPS toxicity in mice having undergone surgical vagotomy (bilateral sub-diaphragmatic with transgastric pyloric dilation) was assessed. Following vagotomy, famotidine had no effect on LPS-induced levels of TNF or IL-6 at 2.5 hours post-LPS injection (Fig. 5 A–D). Stimulating the vagus nerve culminates in acetylcholine release which ultimately mediates inhibits cytokine release by activating α7nAchR on immune competent cells (39, 40). To determine if α7AchR is required for famotidine-dependent inhibition of cytokines, α7AchR knockout mice were treated with famotidine during cytokine storm. Inhibition of proinflammatory cytokine production by famotidine was greatly attenuated in α7AchR KO mice (Fig. 6A–D), which also suffered increased mortality (Fig. 6E). Together these observations show that the anti-inflammatory effects of famotidine are dependent upon activation of the vagus nerve inflammatory reflex which requires α7AchR.

## Discussion

The results of the present study provide evidence that in the setting of endotoxin-induced cytokine storm famotidine activates a potent suppression of pro-inflammatory cytokines leading to improved survival. Mechanistically, famotidine inhibits cytokine release via vagus nerve signaling, as evidenced by the observation of increased vagus nerve activity following famotidine administration and loss of anti-inflammatory activity following vagotomy. This anti-inflammatory activity depends on the α7AChR, and based upon the results of previous study, are attributable to the inhibition of α7AChR positive macrophage pro-inflammatory cytokine release (41). A prior study has also identified the existence of anti-inflammatory neural pathways operating via efferent projections from the dorsal motor nucleus (DMN) directly to visceral organs, e.g., the intestines, which could reasonably explain these observations(42). In support of this hypothesis, direct administration of famotidine, or the structurally related H2R antagonist tiotidine into the ventricular system in close proximity to the DMN, reduced LPS-induced pro-inflammatory cytokine release at one tenth the amount required when delivered via the intraperitoneal route. Future investigation will need to focus on the effects of famotidine on the DMN as well as its widely ranging interconnections to other locations within the brain to explore this possibility further.

In contrast, both cimetidine and ranitidine, relatively selective lower potency H2R antagonists, were ineffective even when administered ICV, suggesting strongly that the anti-inflammatory activity of famotidine (and tiotidine) occur via an off-target effect. This discrepancy cannot be explained simply by the tissue concentrations of cimetidine or ranitidine being below the IC_50_ of the H2R. Specifically, direct observation has shown that an ~ 3-fold increase above the IC_50_ is required to fully antagonize H2R in vivo (43). Although systemic concentrations of cimetidine administered would not likely have reached a 3-fold higher than IC_50_ concentration (~1.5 μM (13)), the maximum dose administered directly into the small CSF volume of the mouse (~35 μL (44)) would exceed the IC_50_ by a factor of ~5000. Similar considerations apply to ranitidine, with an IC_50_ for H2R of ~0.2 μM.

Two additional activities of H2R antagonists have been reported which could directly affect inflammatory processes. First, famotidine has documented powerful antioxidant effects in vitro, particularly for scavenging nitric oxide(18), the hydroxyl radical (20), and myeloperoxidase-catalyzed reactions (22) which could serve to directly reduce inflammation. However, this effect cannot explain the current observations as cimetidine and ranitidine also are potent antioxidants, which is explained a critical sulfur atom as a component of the molecular structures (19). Additionally, antioxidative effects would not explain the dependency of an intact vagus nerve on the observed anti-inflammatory effects. A second potentially relevant biological activity is the documented weak anti-cholinesterase activity of H2R antagonists (45) which could theoretically lead to direct activation of α7nACh and thereby inhibiting the release of pro-inflammatory mediators. However, similar to the case of antioxidant activity, the fact that an intact vagus nerve is required for anti-inflammatory activity as well as the observation that both cimetidine and ranitidine possess anticholinesterase activity rule this out as a possible explanation. Further study will be required to evaluate these possibilities.

It should be noted that the current study has specifically addressed the activity of famotidine in the setting of severe inflammation caused by a model of cytokine storm and therefore the relevance of the activation of the inflammatory reflex under conditions of milder inflammatory conditions, e.g., mild to moderate symptomatic COVID-19, is currently unclear. Considering this uncertainty, future clinical study to evaluate famotidine’s potential beneficial effects should focus on documenting modulation of pro-inflammatory cytokines in the setting of severe COVID-19, as the inflammatory reflex may be of less importance in mildly symptomatic disease.

Finally, the ability of famotidine to activate the inflammatory reflex suggests that famotidine may offer therapeutic benefit in a wide variety of disease processes driven by inflammation. Direct electrical stimulation of the vagus nerve, and thereby activation of the inflammatory reflex, has shown benefit in diverse preclinical models (39) as well as clinical trials, e.g., drug resistant rheumatoid arthritis (46) or inflammatory bowel disease (47). Famotidine, a well-tolerated oral drug, could offer an additional method of activating the inflammatory reflex to reduce pro-inflammatory cytokine generation and resultant tissue damage generated by diverse disease processes.

## Supplementary Material

1

## Data Availability

The datasets analyzed during the current study are available from the corresponding author on reasonable request.

## Abbreviations

FM: famotidine
CM: cimetidine
HMGB1: high mobility group box 1
SARS-CoV-2: severe acute respiratory syndrome coronavirus 2
ICV: intracerebroventricular
LPS: lipopolysaccharide
IP: intraperitoneal
KO: knockout
H2R: histamine 2 receptor
VGX: vagotomy
ELISA: Enzyme-linked immunosorbent assay
PBS: phosphate buffered saline
TNF: Tumor necrosis factor
IL: Interleukin
CXCL1: chemokine (C-X-C motif) ligand 1
α7nAChR: alpha 7 nicotinic acetylcholine receptor
WT: Wild type
ANOVA: Analysis of variance
