# Supplementary material for: Famotidine activates the vagus nerve inflammatory reflex to attenuate cytokine storm
Source: Res Sq. 2022 Apr 11:rs.3.rs-1493296. Preprint. [Version 1] doi: 10.21203/rs.3.rs-1493296/v1 (PMC9016653; doi:10.21203/rs.3.rs-1493296/v1)
Supplement: 1 [file NIHPPRS1493296V1-supplement-1.pdf]

**Supplementary figures.**

**Supplement figure 1. Intraperitoneal administration of famotidine did not significantly alter IL-1β or CXCL1 levels at 2.5 or 6 hours post LPS exposure.**

**A-D.** Male C57BL/6 mice, 8-12 weeks of age, were injected with LPS (7 mg/kg) with or without famotidine (FM, 0.4 or 4 mg/kg, in 100 µl volume), intraperitoneally 30 min before LPS injection. Mice were euthanized 2.5 hours after LPS or FM administration. Serum and spleen IL-1β and CXCL1 were measured (N=10 mice per group), as well as serum IL-6 (N=5 mice per group). **E-H.** Mice received an IP injection of famotidine or vehicle (0.04 or 0.4 mg/kg, in 5 µl volume) 30 min before LPS injection. LPS was administered IP at 7 mg/kg. Mice were euthanized 6 hours post-LPS injection. N=5 for normal group. N=13-14 for others.

**Supplement figure 2. Effects of other histamine 2 receptor antagonists.**

**A-D**. Male C57BL/6 mice had ICV injection of PBS or tiotidine (0.04 or 0.4 mg/kg in 5 µl volume) 30 min before LPS. LPS was administered IP at 7 mg/kg. Mice were euthanized 2.5 hours post-LPS injection and levels of serum and spleen TNF and IL-6 were measured. N=5 for normal group. N=4 for tiotidine 4 mg/kg group and N=8 for other groups. $^*P<0.02$, $^{***}P<0.0001$. **E-K**. Male C57BL/6 mice, 8-12 weeks of age, received cimetidine or PBS (vehicle) via ICV injection 30 min prior to LPS (IP, 7mg/kg). Mice were euthanized 2.5 hours later and serum and spleen cytokines were measured. N=3-6 for normal group, N=9 for other groups. **L-O**. Male C57BL/6 mice had ICV injection of PBS, ranitidine (0.4 mg/kg in 5 µl volume) 30 min before LPS. LPS was administered IP at 7 mg/kg. Mice

were euthanized 2.5 hours post-LPS injection and levels of serum and spleen TNF and IL-6 were measured. N=5 for normal group, N=8 for LPS + PBS and N=10 for LPS + ranitidine group. *$P \leq 0.05$.

**P.** Male C57BL/6 mice, 8-12 weeks old, were injected with LPS (6mg/kg, IP). CM or PBS (4 mg/kg, in 100 µl volume) were injected intraperitoneally twice a day for 3 days, survival was monitored for 2 weeks. N=10 mice (LPS+ cimetidine) or 30 (LPS +PBS) per group.

**Supplement figure 3. Famotidine (ICV administration) attenuates lipopolysaccharide (LPS)-induced IL-6 release in mice at 6 hours post-LPS administration.**

A-B. Male C57BL/6 mice, 8-12 weeks of age, were injected with LPS (7 mg/kg) with or without famotidine (FM, 0.04 or 0.4 mg/kg) administered ICV at 30 min before LPS injection. Mice were euthanized 2.5 hours after LPS administration. Serum IL-6 and IL-1β were measured. N=5 mice per group for normal, 4 or 8 for LPS alone, 10-15 for other groups. *P=0.008.
